# Hepatitis B Core-Related Antigen Point-of-Care Tests as a Risk Stratification Tool for Treatment Eligibility: Experience From Kenya

**DOI:** 10.1093/ofid/ofaf125

**Published:** 2025-03-06

**Authors:** Louise O Downs, Dorcas Okanda, Oscar Chirro, Mwanakombo Zaharani, Benson Safari, Nadia Aliyan, Monique I Andersson, Yasuhito Tanaka, Anthony O Etyang, Yusuke Shimakawa, George Githinji, Philippa C Matthews

**Affiliations:** Nuffield Department of Medicine, University of Oxford, Oxford, UK; KEMRI-Wellcome Trust Research Programme, Kilifi, Kenya; KEMRI-Wellcome Trust Research Programme, Kilifi, Kenya; KEMRI-Wellcome Trust Research Programme, Kilifi, Kenya; KEMRI-Wellcome Trust Research Programme, Kilifi, Kenya; KEMRI-Wellcome Trust Research Programme, Kilifi, Kenya; Kilifi County Hospital, Kilifi, Kenya; Radcliffe Department of Medicine, University of Oxford, Oxford, UK; Oxford University Hospitals, Oxford, UK; Department of Gastroenterology and Hepatology, Faculty of Life Sciences, Kumamoto University, Kumamoto, Japan; Pasteur International Unit at Kumamoto University, National Center for Global Health and Medicine, Kumamoto, Japan; Nuffield Department of Medicine, University of Oxford, Oxford, UK; KEMRI-Wellcome Trust Research Programme, Kilifi, Kenya; Pasteur International Unit at Kumamoto University, National Center for Global Health and Medicine, Kumamoto, Japan; Institut Pasteur, Université Paris Cité, Unité d'Épidémiologie des Maladies Émergentes, Paris, France; KEMRI-Wellcome Trust Research Programme, Kilifi, Kenya; Department of Biochemistry and Biotechnology, Pwani University, Kilifi, Kenya; The Francis Crick Institute, London, UK; Division of Infection and Immunity, University College London, London, UK; University College London Hospital, London, UK

**Keywords:** hepatitis B core-related antigen, HBV, liver disease, point-of-care diagnostics, treatment

## Abstract

We undertook a point-of-care test for hepatitis B core-related antigen in adults with hepatitis B virus in Kilifi, Kenya. A positive test identified all individuals with a hepatitis B viral load >200 000 IU/mL and who were hepatitis B e antigen positive. It also correlated with a higher alanine aminotransferase (ALT) level (*P* = .03), raised aspartate transaminase-to-platelet ratio index (APRI) (*P* < .001), and higher elastography scores (*P* = .03).

The 2024 Global Hepatitis Report highlights significant morbidity and mortality due to chronic hepatitis B virus infection (CHB), with the number of people dying from CHB rising from 820 000 in 2019 to 1.1 million in 2022, disproportionately affecting populations in the World Health Organization African Region (WHO-AFRO) [1].

One of the most significant barriers to HBV elimination in this region is that only 0.2% of people with HBV (PWHB) are on treatment [1], and tests for risk stratification are often inaccessible and unaffordable. New 2024 WHO HBV management guidelines simplify assessment, but still recommend alanine aminotransferase (ALT) measurement or aspartate transaminase (AST)-to-platelet ratio index (APRI) calculation [2], along with HBV viral load (VL) measurement to determine long-term treatment eligibility. HBV VL measurement and/or hepatitis B e antigen (HBeAg) assessment are also recommended to determine eligibility for peri-partum antiviral prophylaxis [3].

Point-of-care tests (POCTs) offfer an attractive alternative to these laboratory assays, allowing real-time feedback, potentially improving retention in clinical care and viral suppression rates [4]. Currently, liver stiffness measurement using elastography is the only validated POCT for assessing liver health for PWHB; however, the hardware is prohibitively expensive. Current POCTs for HBeAg have poor sensitivity [5, 6], and HBV VL measurements require a laboratory platform like Gene Xpert (Cepheid Inc., Sunnyvale, CA, USA).

Hepatitis B core-related antigen (HBcrAg) correlates with peripheral HBV DNA and HBeAg status in untreated individuals [7] and could be an alternative to HBV VL quantification in low-resource settings [8]. HBcrAg has previously only been available as a chemiluminescent assay (CLEIA). A new HBcrAg POCT performed well in The Gambia as a surrogate for HBV VL, with a positive test being 91.4% sensitive and 86.3% specific for detecting HBV VL >200 000 IU/mL [9]. Using the laboratory HBcrAg CLEIA as a reference, the limit of detection of the HBcrAg POCT was around 4.3 logU/mL. The POCT has not yet been assessed elsewhere in the general adult population with CHB in WHO-AFRO countries, and performance has not been evaluated against ALT or liver fibrosis. We set out to evaluate the HBcrAg POCT in PWHB in Kilifi, Kenya, considering (i) the ease of testing; (ii) its relationship with ALT, HBeAg status, HBV VL, and elastography; and (iii) its contribution to determining treatment eligibility.

## METHODS

PWHB were recruited through the STRIKE-HBV study at KEMRI-Wellcome Trust Research Programme (KWTRP), which identified 102 nonpregnant adults from Kilifi County, Kenya, as previously described [10]. Positive HBsAg POCT results were confirmed with an enzyme-linked immunoassay (ELISA; Murex HBsAg 3, Diasorin). The cohort was 60% female, median age (interquartile range [IQR]) 37 (28–49) years, and 2 people were known to have HIV infection. Testing for hepatitis C virus infection was not done due to low prevalence [11] and lack of treatment availability. Testing for hepatitis D virus was not available.

Transient elastography was undertaken to assess liver health at recruitment (FibroScan; Echosens, Paris, France), and blood samples were drawn and transported to the research laboratory within 2–3 hours of collection, spun to separate serum, and frozen in aliquots at −80°C. Routine laboratory markers were measured in a validated diagnostic clinical laboratory at KWTRP, including ALT, AST (Ilab Aries), and platelets (AcT 5diff CP, Beckman Coulter) for APRI score calculation.

HBeAg was tested at the KWTRP research laboratory using CLEIA (Ig Biotechnology, CA, USA), HBV VL was tested at Oxford University Hospitals, UK, using the Abbott Alinity m HBV Assay (IL, USA). The HBcrAg POCT (ESPLINE [RUO], Fujirebio, Japan) was shipped from Kumamoto University, Japan, stored at room temperature, and undertaken retrospectively on 50-µL defrosted serum in the KWTRP laboratories as per the manufacturer's instructions [9]; no specific training was required. The testing was performed in 3 batches by clinical and laboratory staff blinded to patient details including HBV VL and HBeAg status. Positive and negative controls were provided and undertaken once before each batch testing.

Liver health thresholds and treatment eligibility criteria were taken from the 2024 WHO HBV guidelines [2] as follows: elastography score >7.0 kPa = significant fibrosis; >12.5 kPa = cirrhosis; APRI >0.5 = significant fibrosis; >1.0 = cirrhosis. Those with the following were defined as treatment eligible: (i) elastography score >7 kPa; OR (ii) APRI score >0.5; OR (iii) HBV DNA >2000 IU/mL AND ALT > upper limit of normal (ULN; 19 IU/L for women, 30 IU/L for men), OR HIV coinfected. In the untreated population, we first investigated whether ALT alone could identify those who were treatment eligible, then evaluated the contribution of the HBcrAg POCT. We calculated sensitivity, specificity, positive predictive value, and negative predictive value for how well these tests identify those eligible for treatment based on WHO guidelines (Supplementary Figure 1). We chose to use WHO guidelines as these have been recently updated and include specific recognition of service delivery in resource-limited settings, and will thus infrom practice in Kenya and elsewhere in the WHO African region. Statistical analysis was done using R, version 4.2.0.

## RESULTS

HBcrAg POCT was positive in 14/102 PWHB (14%)—with a positivity rate of 4/61 (7%) in women and 10/41 (24%) in men (*P* = .01). Those with a positive POCT were younger (median age, 28 years compared with 38 years with a negative POCT; *P* = .03). There was no difference in current antiviral treatment status between those testing POCT positive or negative (Table 1). Laboratory personnel reported that the POCT was easy to perform. The test turnaround time was ∼40 minutes.

**Table 1.** Characteristics of 102 Adults With Chronic HBV Infection in Kilifi, Kenya, Assessed With a POCT for HBcrAg

Characteristic (All Participants)	n = 102	Negative HBcrAg POCT n = 88	Positive HBcrAg POCT n = 14	*P* Value^a^
Sex, No. (%)	102	…	…	**.010**
Female	61	57/61 (93)	4/61 (7)	…
Male	41	31/41 (76)	10/41 (24)	…
Age, median (IQR), y	102	38 (30–49)	28 (27–39)	**.03**
Unknown	1	1/1 (100)	0/1 (0)	…
On antiviral therapy, No. (%)	102	…	…	.8
Yes	27	24/27 (89)	3/27 (11)	…
No	75	64/75 (85)	11/75 (15)	…
Characteristic (untreated group)	n = 75	Negative HBcrAg POCT n = 64	Positive HBcrAg POCT n = 11	*P* Value^a^
HBV DNA, median (IQR), log_10_ IU/mL	74	2.68 (1.56–3.45)	6.05 (3.19–7.03)	**<.001**
HBV DNA group, No. (%)	75	…	…	**<.001**
<20 log_10_ IU/mL	13	12/13 (92)	1/13 (8)	…
20–2000 log_10_ IU/mL	36	34/36 (94)	2/36 (6)	…
2000–20 000 log_10_ IU/mL	15	14/15 (93)	1/15 (7)	…
20 000–200 000 log_10_ IU/mL	3	3/3 (100)	0/3 (0)	…
>200 000 log_10_ IU/mL	7	0/7 (0)	7/7 (100)	…
Unknown	1	1/1 (100)	0/1 (0)	…
ALT, median (IQR), U/L	75	26 (19–35)	58 (31–67)	**.006**
ALT > ULN, No. (%)	75	…	…	.7
Yes	48	40/48 (83)	8/48 (17)	…
No	27	24/27 (89)	3/27 (11)	…
Elastography score, median (IQR), kPa	66	4.25 (3.50–5.55)	6.65 (3.50–8.98)	.2
Unknown, No. (%)	9	6/9 (67)	3/9 (33)	…
Liver health FibroScan, No. (%)	75	…	…	**.03**
Normal (≤7 kPa)	57	52/57 (91)	5/57 (9)	…
Significant fibrosis (>7−≤12.5 kPa)	8	6/8 (75)	2/8 (25)	…
Cirrhosis (>12.5 kPa)	1	0/1 (0)	1/1 (100)	…
Unknown	9	6/9 (67)	3/9 (33)	…
APRI score, median (IQR)	74	0.29 (0.22–0.39)	0.57 (0.34–0.86)	**.003**
Unknown, No. (%)	1	1/1 (100)	0/1 (0)	**…**
Liver health APRI, No. (%)	75	…	…	**<.001**
Normal (<0.5)	60	57/60 (95)	3/60 (5)	…
Significant fibrosis (0.5–1)	11	5/60 (45)	6/60 (55)	…
Cirrhosis (>1)	3	1/3 (33)	2/3 (67)	…
Unknown	1	1/1 (100)	0/1 (0)	…
HBeAg, No. (%)	75	…	…	**<.001**
Positive	7	0/7 (0)	7/7 (100)	…
Negative	68	64/68 (94)	4/68 (6)	…

Data are presented to show the percentage in each category, adding up to 100% in each row to allow comparison of characteristics between those testing HBcrAg POCT positive vs negative. Significant *P*-values (<.05) are in bold.

Abbreviations: ALT, alanine aminotransferase; APRI, aspartate transaminase-to-platelet ratio index; HBcrAg, hepatitis B core-related antigen; HBeAg, hepatitis B e antigen; IQR, interquartile range; POCT, point-of-care testing; ULN, upper limit of normal.

^a^Pearson chi-square test; Wilcoxon rank-sum test; Fisher exact test.

### Analysis in the Untreated Population

At cohort entry, 75 PWHB were untreated (Table 1), and 27 were on nucleoside analogue (NA) therapy (Supplementary Table 1). Among the 75 individuals not receiving treatment (42 female and 33 male), none was HIV coinfected. The median age (IQR) was 40 (27–49) years; 11/75 individuals (15%) had a positive HBcrAg POCT. This group had a significantly higher median HBV DNA level than those with a negative POCT (6.05 log_10_ IU/mL vs 2.7 log_10_ IU/mL, respectively; *P* < .001) (Table 1). To identify those with HBV DNA >200 000 IU/mL, HBcrAg POCT had a sensitivity of 100% (7/7) (Supplementary Figure 2A) and a specificity of 94% (64/68). All those who had HBV DNA >200 000 IU/mL also tested HBeAg positive (Table 1), and all those HBeAg positive were also HBcrAg POCT positive (Supplementary Figure 2B).

### Comparison With Liver Health Markers

A positive HBcrAg POCT was significantly associated with a higher median ALT than a negative test (58 IU/L vs 26 IU/L; *P* = .006) (Table 1). Those with a positive POCT also had higher median APRI scores (0.57 vs 0.29; *P* = .003) and were more likely to meet APRI criteria for significant fibrosis or cirrhosis (8/11 [73%] vs 6/63 [10%], respectively; *P* < .001) (Table 1;  Supplementary Figure 2C). Individuals with a positive HBcrAg POCT were more likely to meet elastography criteria for significant fibrosis or cirrhosis than those with a negative POCT (3/11 [27%] vs 6/64 [9%] with a negative test; *P* = .03) (Supplementary Figure 2D), but there was no significant difference in median elastography score between those with a positive or negative HBcrAg POCT (Table 1).

### Review of Treatment Criteria

In this currently untreated group, 27/75 (36%) people met treatment criteria as defined by the WHO guidelines (Supplementary table 2). The sensitivity of abnormal ALT alone for identifying treatment eligibility was 85% (23/27), compared with 33% (9/27) for a positive HBcrAg POCT alone. Defining treatment eligibility as either an abnormal ALT OR a positive HBcrAg POCT increased the sensitivity to 89% (24/27), identifying 1 extra person as treatment eligible compared with either test alone (Supplementary Table 2 and Supplementary Figure 3). The extra individual identified had a high HBV VL of 6.1 log_10_ IU/mL and a FibroScan score of 10.1 kPa, suggesting some existing liver fibrosis, a risk of progressive chronic liver disease, and a potential transmission risk. The improved sensitivity was to the detriment of specificity, but as per 2024 WHO guidelines, overtreating is preferential to missing those who need treatment [2].

The positive predictive value (PPV) of the HBcrAg POCT alone for treatment eligibility was 82%, but the negative predictive value (NPV) was only 28%. Defining treatment eligibility as either a positive HBcrAg POCT OR an abnormal ALT reduced the PPV to 47% but improved the NPV to 88%, indicating an 88% certainty that the individual did not meet treatment criteria if both tests were normal (Supplementary Table 2).

## DISCUSSION

This is the first report directly comparing the HBcrAg POCT with ALT, markers of liver fibrosis, and HBeAg status. Here, a positive HBcrAg POCT was strongly associated with liver inflammation by ALT and with fibrosis/cirrhosis by both FibroScan and APRI scores. However, as expected, the HBcrAg POCT alone had a lower sensitivity for identifying those meeting WHO treatment criteria than ALT alone (33% vs 85%). Addition of the POCT to ALT did identify 1 extra person as treatment eligible, and the combination of tests improved the sensitivity of either test alone; however, further assessment is needed to see if this benefit translates to larger populations. Although the NPV of both tests combined was 88%, using this approach to make treatment decisions would still miss 12% of people at risk of liver disease progression in this population.

In this cohort, a positive HBcrAg POCT was 100% sensitive at identifying a sample that was laboratory positive for HBeAg, performing much better than currently available HBeAg POCTs, which have sensitivities ranging from 30% to 62% [5, 6]. In this study, a positive HBcrAg POCT was 100% sensitive at identifying those with HBV VL >200 000 IU/mL, which is higher than reported from the Gambia [9]. This POCT is therefore of potential clinical utility in identifying individuals eligible for perinatal antiviral prophylaxis. This is the first time this POCT has been evaluated in East Africa, where circulating HBV genotypes differ from those in West Africa.

This HBcrAg POCT is quick and could be undertaken without specific training. It has a relatively low production cost (<$5 per test) compared with HBV VL, which varies in price across WHO-AFRO up to $62/test [1], and HBeAg, which can be up to $40/test for the laboratory ELISA [12, 13], assuming these tests are actually available. ALT is relatively low cost (<$10) and available in much of WHO-AFRO, so it continues to be a good method of liver health assessment in the absence of other tests. Further implementation research is needed to determine whether inclusion of the HBcrAg POCT alongside ALT could enhance linkage to care and improve decentralization of clinical assessment and how its impact and cost-effectiveness varies by setting.

The number of PWHB included in this study was small, from a specific geographical region, and did not include children/adolescents or pregnant women. We did not have access to a HBeAg POCT or gold standard method of assessing liver disease such as cross-sectional imaging or liver biopsy.

## CONCLUSIONS

HBcrAg POCT correlates strongly with several liver disease markers in PWHB. However, it does not perform better than ALT at identifying those with abnormal elastography or APRI scores, so it should be used as an adjunct rather than a standalone. It could provide a low-cost alternative to some otherwise unavailable, unaffordable diagnostics; further evaluation is required in different settings.

## Supplementary Material

ofaf125_Supplementary_Data

## Supplementary Data

Supplementary materials are available at *Open Forum Infectious Diseases* online. Consisting of data provided by the authors to benefit the reader, the posted materials are not copyedited and are the sole responsibility of the authors, so questions or comments should be addressed to the corresponding author.
