# Supplementary material for: Hepatitis B Core-Related Antigen Point-of-Care Tests as a Risk Stratification Tool for Treatment Eligibility: Experience From Kenya
Source: Open Forum Infect Dis. 2025 Mar 6;12(3):ofaf125. doi: 10.1093/ofid/ofaf125 (PMC11923541; doi:10.1093/ofid/ofaf125)
Supplement: ofaf125_Supplementary_Data [file ofaf125_supplementary_data.docx]

SUPPLEMENTARY MATERIAL

**Hepatitis B core-related antigen (HBcrAg) point-of-care tests as a risk stratification tool: experience from Kenya**

CONTENTS:

**Supplementary Table 1:** **Characteristics of 27 adults living with chronic hepatitis B virus infection in Kilifi, Kenya, taking nucleoside analogue therapy assessed with a point of care test (POCT) for Hepatitis B core-related antigen (HBcrAg).**

**Supplementary Table 2: The number of people living with hepatitis B virus infection in Kilifi, Kenya meeting treatment criteria (defined as Fibroscan score >7kPa, or APRI score >0.5, or the combination of raised ALT AND HBV DNA >2000 IU/ml) compared with how many of these people would have been identified using ALT alone, HBcrAg-POCT alone or by either test being positive**

Supplementary Figure 1: Calculation of sensitivity, specificity, positive and negative predictive values of i) Abnormal ALT alone, ii) Positive HBcrAg POCT alone, and iii) Either abnormal ALT OR positive HBcrAg POCT in a population of adults living with chronic hepatitis B infection in Kilifi, Kenya.

**Supplementary Figure 2: Relationship between clinical characteristics and results of HBcrAg point-of-care test (POCT) in adults living with hepatitis B virus infection (HBV) in Kilifi, Kenya**

**Supplementary figure 3: Venn diagram showing numbers of untreated adults living with hepatitis B infection who would be eligible for nucleoside analogue therapy based on different criteria**

| **Characteristic (treated group)** | **N** | **Negative HBcrAg POCT**  **N = 24*^1^*** | **Positive HBcrAg POCT. N = 3*^1^*** | **p-value***^2^* |
| --- | --- | --- | --- | --- |
| **Sex** | 27 |  |  | 0.2 |
| Female | 19 | 18/19 (95%) | 1/19 (5%) |  |
| Male | 8 | 6/8 (75%) | 2/8 (25%) |  |
| **Age in years, median (IQR)** | 26 | 36 (30, 45) | 34 (33, 49) | >0.9 |
| Unknown | 1 | 1/1 (100%) | 0/1 (0%) |  |
| **HBV DNA (log10 IU/ml), median (IQR)** | 27 | 2.21 (0.00, 2.27) | 2.14 (0.00, 2.27) | >0.9 |
| **HBV DNA Group (log_10_ IU/ml)** | 27 |  |  | >0.9 |
| <20 | 11 | 10/11 (91%) | 1/11 (9%) |  |
| 20-2000 | 16 | 14/16 (88%) | 2/16 (12%) |  |
| 2000-20,000 | 0 | 0 | 0 |  |
| 20,000-200,000 | 0 | 0 | 0 |  |
| >200,000 | 0 | 0 | 0 |  |
| **ALT (U/L), median (IQR)** | 27 | 28 (22, 32) | 35 (17, 77) | 0.5 |
| **ALT >ULN** | 27 |  |  | >0.9 |
| Yes | 18 | 16/18 (89%) | 2/18 (11%) |  |
| No | 9 | 8/9 (89%) | 1/9 (11%) |  |
| **Elastography score (kPa), median (IQR)** | 25 | 5.05 (4.00, 6.00) | 5.90 (3.50, 18.20) | 0.6 |
| Unknown | 2 | 2/2 (100%) | 0/2 (100%) |  |
| **Liver Health Fibroscan** | 27 |  |  | 0.15 |
| Normal (≤7kPa) | 21 | 19/21 (90%) | 2/21 (10%) |  |
| Fibrosis (>7 - ≤12.5kPa) | 3 | 3/3 (100%) | 0/3 (0%) |  |
| Cirrhosis (>12.5kPa) | 1 | 0/1 (0%) | 1/1 (100%) |  |
| Unknown | 2 | 2/2 (100%) | 0/2 (0%) |  |
| **APRI Score, median (IQR)** | 27 | 0.25 (0.20, 0.34) | 0.45 (0.19, 1.54) | 0.4 |
| **Liver health APRI** | 27 |  |  | 0.3 |
| Normal (<0.5) | 24 | 22/24 (92%) | 2/24 (8%) |  |
| Fibrosis (0.5 – 1) | 1 | 1/1 (100%) | 0/1 (0%) |  |
| Cirrhosis (>1) | 2 | 1/2 (50%) | 1/2 (50%) |  |
| Unknown | 0 | 0 | 0 |  |
| **HBeAg** | 27 |  |  | >0.9 |
| Positive | 0 | 0 | 0 |  |
| Negative | 26 | 23/26 (88%) | 3/26 (12%) |  |
| Unknown | 1 | 1/1 (100%) | 0/1 (0%) |  |
| *^1^*n (%); Median (Q1, Q3) | | | | |
| *^2^*Fisher's exact test; Wilcoxon rank sum test; Wilcoxon rank sum exact test | | | | |

**Supplementary Table 1:** **Characteristics of 27 adults living with chronic hepatitis B virus infection (HBV) in Kilifi, Kenya, taking nucleoside analogue therapy assessed with a point of care test (POCT) for Hepatitis B core-related antigen (HBcrAg). IQR – interquartile range; ALT – alanine aminotransferase; ULN – upper limit of normal; kPa – kilopascals; APRI – aspartate to platelet ratio index; HBeAg – hepatitis B ‘e’ antigen.**

|  | 27/75 (36%) met treatment criteria | Identified by abnormal ALT alone | Identified by positive HBcrAg-POCT alone | Identified by abnormal ALT OR positive HBcrAg-POCT |
| --- | --- | --- | --- | --- |
| Sensitivity |  | 85% | 33% | 89% |
| Specificity |  | 48% | 96% | 44% |
| PPV |  | 48% | 82% | 47% |
| NPV |  | 85% | 28% | 88% |

**Supplementary Table 2: The sensitivity, specificity, positive predictive value (PPV) and negative predictive value (NPV) for ALT alone, HBcrAg-POCT alone or by either test being positive at identifying individuals** **living with hepatitis B virus infection in Kilifi, Kenya meeting treatment eligibility criteria (defined as Fibroscan score >7kPa, or APRI score >0.5, or the combination of raised ALT AND HBV DNA >2000 IU/ml).** kPa – kilopascals; APRI – Aspartate transaminase-to-platelet ratio index, ALT – Alanine aminotransferase, HBcrAg-POCT – Hepatitis B core-related antigen point-of-care test; WHO – World Health Organisation. **(Calculated as shown in Suppl Fig 1).**

**
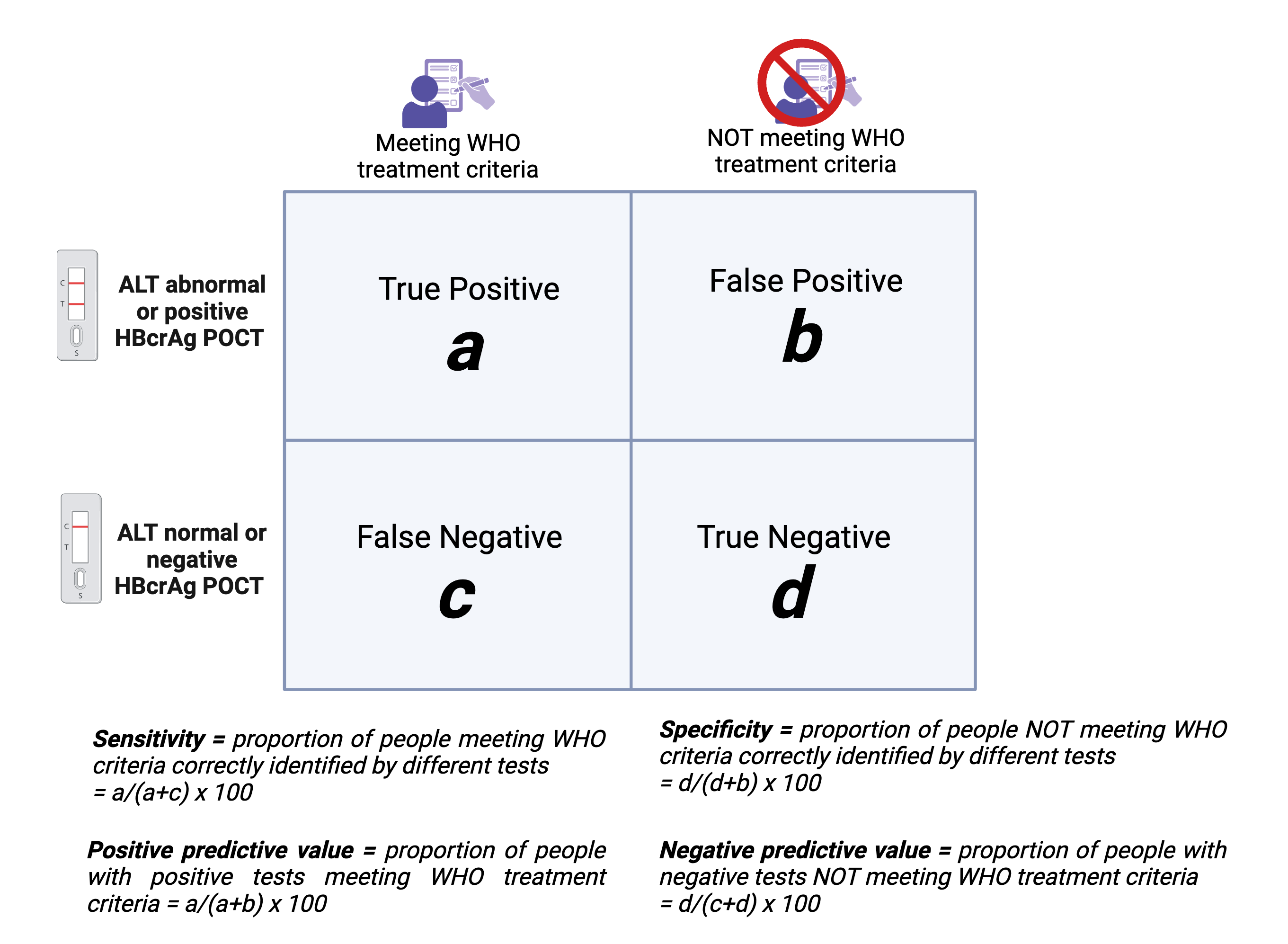
**

**Supplementary Figure 1:** Calculation of sensitivity, specificity, positive and negative predictive values of i) Abnormal ALT alone, ii) Positive HBcrAg POCT alone, and iii) Either abnormal ALT OR positive HBcrAg POCT in a population of adults living with chronic hepatitis B infection in Kilifi, Kenya. Image created with BioRender.com; exported with a license to publish under a paid subscription. WHO – World health Organisation; ALT – alanine aminotransferase, HBcrAg – hepatitis B core related antigen, POCT – point of care test.


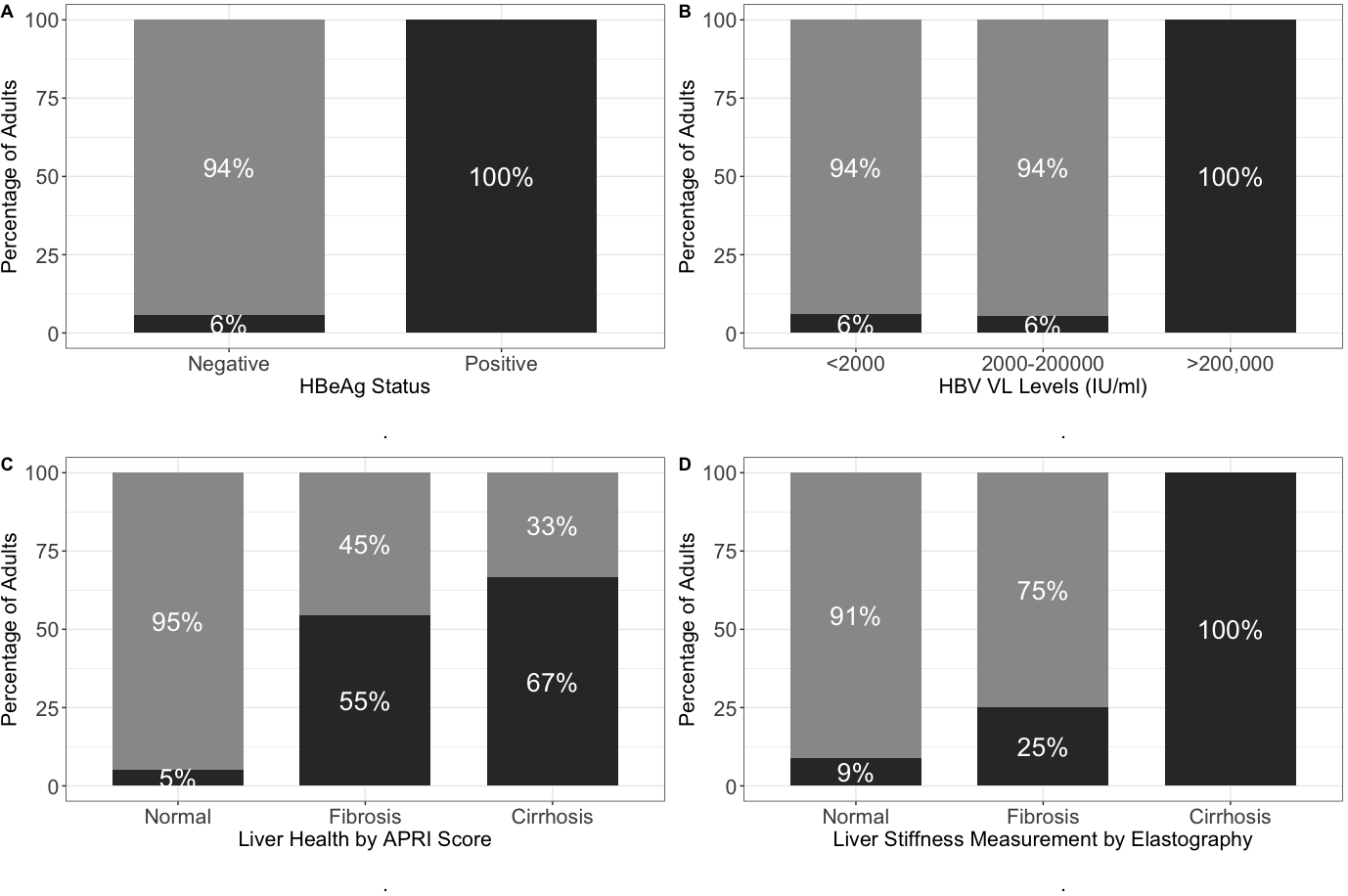

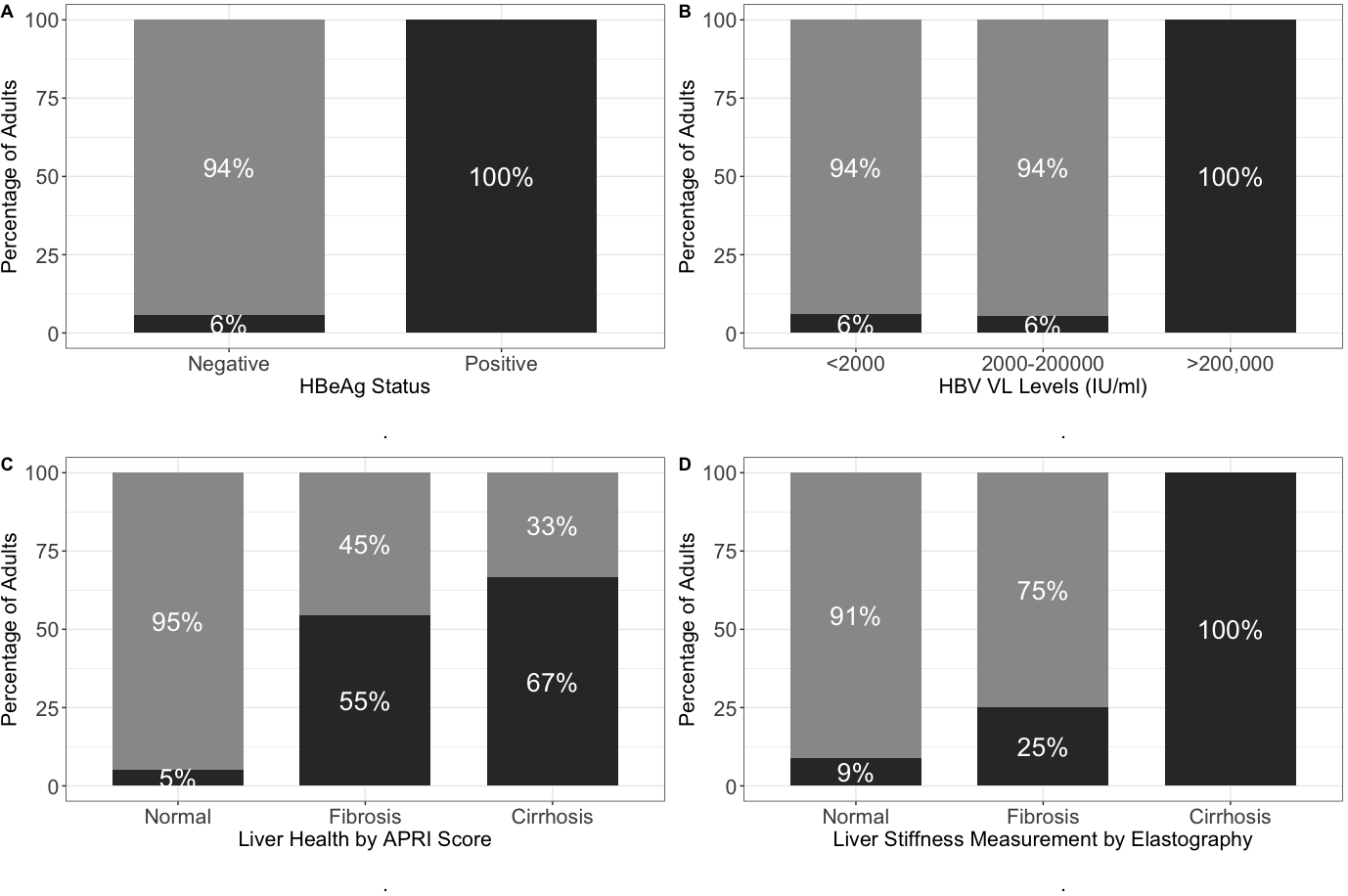

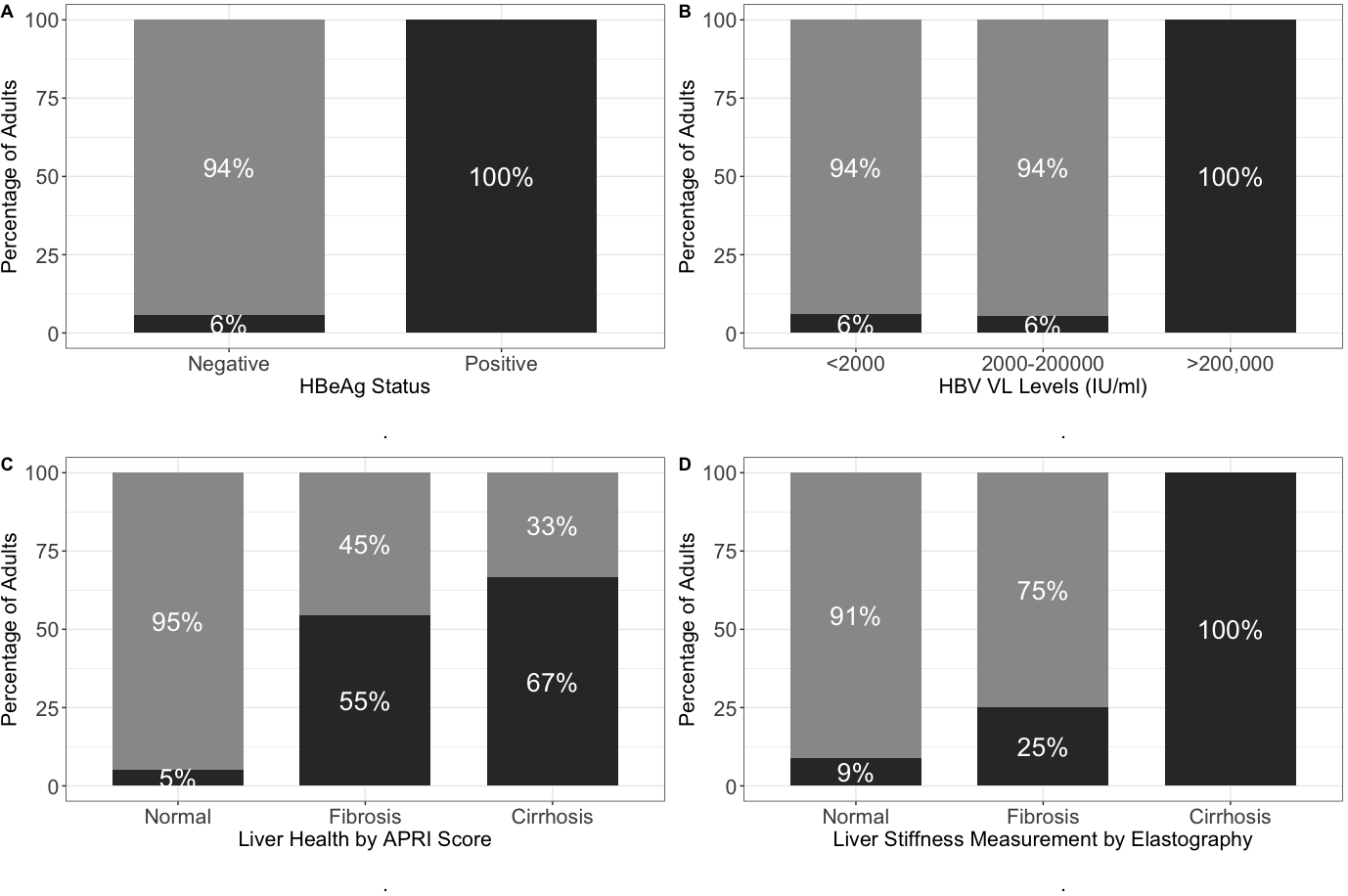


Negative (n=68) Positive (n=7)

<2000 (n=49) 2000-200,000 (n=18) ≥200,000 (n=7)

HBV DNA level (IU/ml)

Normal (n=60) Fibrosis (n=11) Cirrhosis (n=3)

Normal (n=57) Fibrosis (n=8) Cirrhosis (n=1)

Liver Stiffness by Elastography Measurement

HBeAg status

Liver Health by APRI score

A

B

**Supplementary Figure 2: Relationship between clinical characteristics and results of HBcrAg point-of-care test (POCT) in untreated adults living with hepatitis B virus infection (HBV) in Kilifi, Kenya.** A - HBV VL level, B - HBeAg status, C - liver health based on APRI scores, D - Liver stiffness by elastography scores. Each characteristic is split by hepatitis B core-related antigen (HBcrAg) POCT test result. Light grey is those testing HBcrAg POCT negative, dark grey is those testing HBcrAg POCT positive. HBeAg: hepatitis B ‘e’ antigen, APRI: Aspartate transaminase to platelet ratio index. APRI scores: Normal <0.5, Significant fibrosis 0.5 - 1, Cirrhosis >1. Elastography scores (kPa – kilopascals): Normal <7 kPa, Significant fibrosis 7-12.5 kPa, Cirrhosis >12.5 kPa.

**
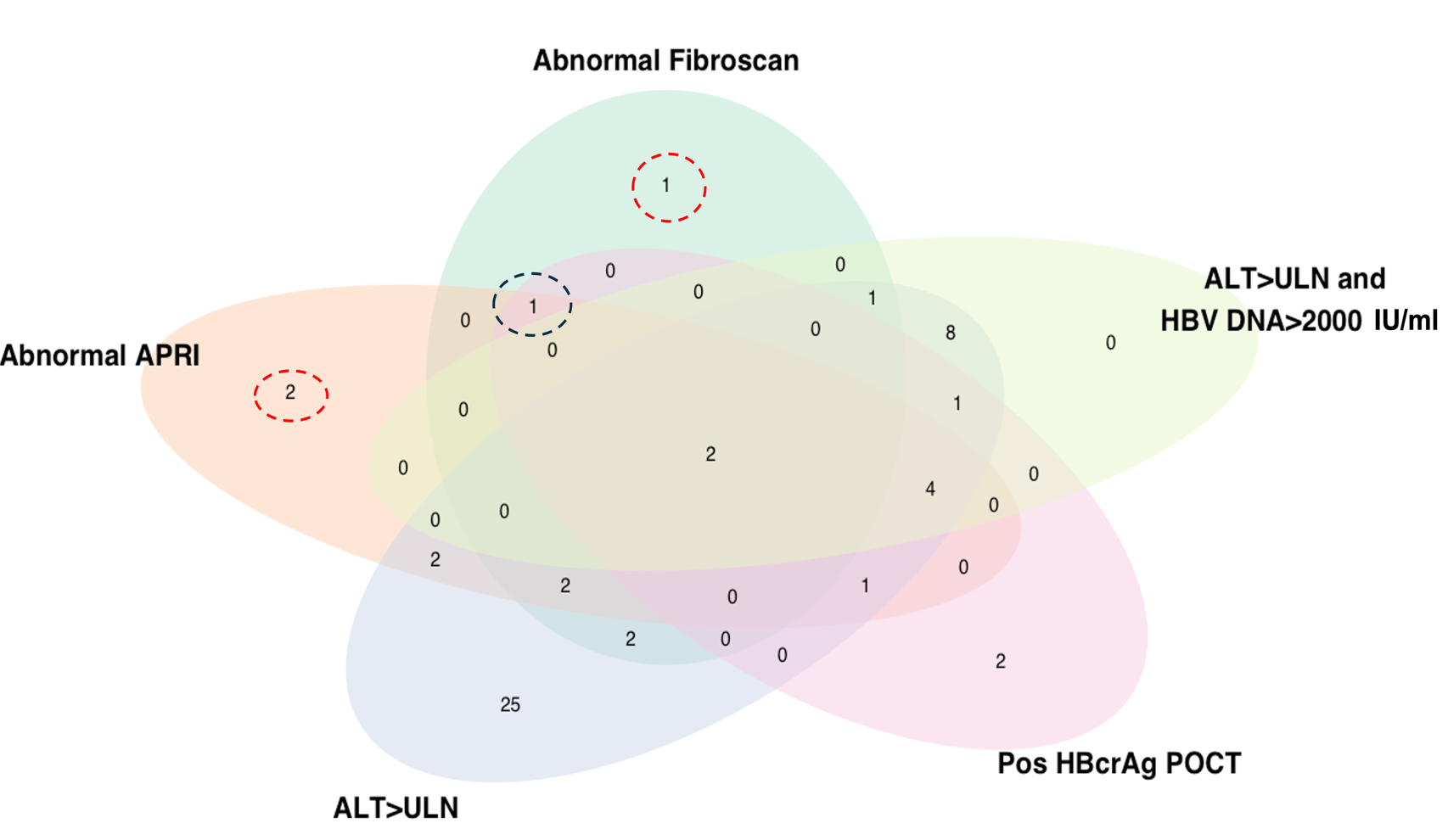
**

**Supplementary figure 3: Venn diagram showing numbers of untreated adults living with hepatitis B infection who would be eligible for nucleoside analogue therapy based on different criteria.** ALT – alanine transferase; APRI – Aspartate transaminase to Platelet Ratio Index; ULN – upper limit of normal; POCT – point of care test; HBcrAg – hepatitis B core related antigen. Black dotted circle indicates the one extra person with abnormal APRI and elastography scores who would have been identified using HBcrAg-POCT but missed using abnormal ALT alone. Red dotted circles indicate the three people with abnormal Fibroscan or APRI scores who would have been missed by both ALT and HBcrAg-POCT.
